# Prenatal diagnosis of rare cloacal exstrophy: A case report

**DOI:** 10.1016/j.amsu.2022.104436

**Published:** 2022-08-17

**Authors:** Febriani, Muhammad Alamsyah Aziz

**Affiliations:** Fetomaternal Division, Department of Obstetrics and Gynecology, Faculty of Medicine, Padjadjaran University, Hasan Sadikin General Hospital Bandung, Indonesia

**Keywords:** Case report, Cloacal exstrophy, Omphalocele, Prenatal diagnosis, Ultrasound

## Abstract

**Introduction and importance:**

Cloacal Exstrophy (CE) is a rare congenital birth defect. A correct prenatal diagnosis of CE is rarely made, even when congenital abnormalities are suspected on prenatal ultrasound examination.

**Case presentation:**

We report a case of CE with an abdominal defect about 5.31 cm in diameter seen below the umbilicus covered by a membrane. It was difficult to identify the left kidney, the bladder, the genital, and the anal dimple in the early third trimester ultrasound. The diagnosis of CE was then confirmed postnatally.

**Clinical discussion:**

Omphalocele and persistent bladder nonvisualization despite normal amniotic fluid volume detected by prenatal ultrasound can be suggestive for CE.

**Conclusion:**

Accurate prenatal diagnosis of CE is important to carry out multidisciplinary management and prenatal counseling to parents.

## Highlights

• Prenatal diagnosis of cloacal exstrophy can be done based on ultrasound findings.
• Omphalocele with bladder nonvisualization can be suggestive for cloacal exstrophy.
• Prenatal diagnosis is important to carry out prenatal counseling to parents.

## 1 Introduction

Cloacal exstrophy (CE) is a rare congenital birth defect with an incidence of 1:200.000 to 1:400.000 live births. As one of the most severe birth defects involving multiple organ systems including neurological, gastrointestinal, musculoskeletal, renal and genitourinary, it requires a multidisciplinary approach [1]. Because of the rarity and the wide range of anatomic variants, prenatal diagnosis of CE is challenging [2].

Here, we present a case of a 26-year-old primigravid woman whose fetus was diagnosed with cloacal exstrophy. This case has been reported in line with the SCARE 2020 guidelines [3].

## 2 Case presentation

A 26-year-old primigravid woman with 33–34 weeks of gestation came to the obstetrics emergency room with complaints of intermittent back pain that radiates to the placenta. During pregnancy, the patient had been treated for hyperemesis gravidarum (HEG). The patient also consumed multivitamins, ferrous sulphate, and folic acid tablet given by an obstetrician during the nine months of pregnancy, and the patient did not show any allergic and/or adverse reactions. There were no history of smoking, alcohol, and recreational drug use. There were no complaints of spotting or bleeding during pregnancy. There were no history of surgery before. Antenatal care (ANC) with an obstetrician in the early third trimester revealed a suspected abdominal wall defect in the form of an omphalocele. The patient denied having family history of congenital malformation. The patient was then referred to our facility.

**Fig. 1:**
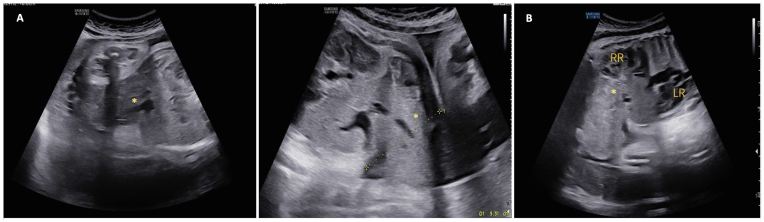
(A) Defect (asterisk) in the midline of the anterior abdominal wall, about 5.31 cm in diameter; (B) Defect (asterisk) at the level of the kidney in the longitudinal section. RR, right renal; LR, left renal.

Physical examination revealed normal vital signs. The patient was diagnosed with G1P0A0 with preterm contractions and suspected congenital abnormalities (omphalocele) of the fetus. The patient was anemic with Hb level of 9,0 g/dL. The cardiotocography was reassuring. Five days before admission to our facility, the patient underwent a detailed ultrasound with the following findings: single intrauterine fetus, 33–34 weeks of gestation (based on biometry), estimated foetal weight (EFW) 2100 g, foetal heart rate (+), and normal thorax 4 chamber view (4CV). There was an abdominal defect about 5.31 cm in diameter (Fig. 1), seen below the umbilicus and on the midline with part of the liver extruding outside the abdominal cavity. The defect was covered by a membrane. It was difficult to identify the left kidney, the bladder, the genital, and the anal dimple. Placenta was found on the posterior uterine corpus with adequate amniotic fluid volume based on single deepest pocket measurement (7.13 cm). Doppler velocimetry was within normal limits.

**Fig. 2:**
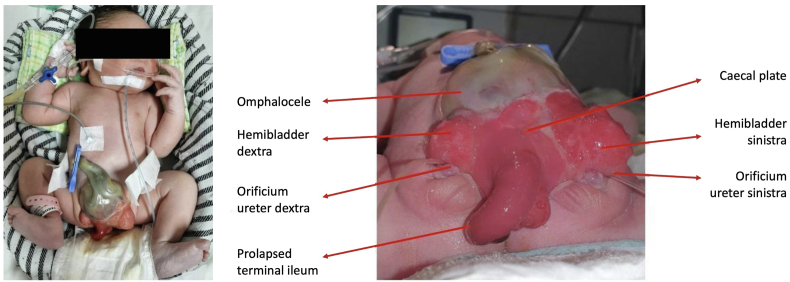
Congenital abnormalities found in the baby with cloacal exstrophy: omphalocele, open bladder in the lower abdomen with the mucosa visible through a triangular fascia defect, protruding ileum, genital malformations, shortened umbilicus to anus distance, and exposed caecal plate.

The patient entered labor on the third day of treatment and the baby was born with a birth weight of 2560 g, body length of 45 cm, ballad score in accordance with 35–36 weeks of gestational age, and multiple congenital abnormalities i.e. omphalocele, an open bladder in the lower abdomen with the mucosa visible through a triangular fascia defect, prominent ileum, genital malformations, and a shortened distance between umbilicus and anus (Fig. 2). The patient's postpartum hemoglobin level decreased to 7.7 g/dL. She received a blood transfusion. The hemoglobin level increases to 9.6 g/dL and she had a good recovery. She was discharged 5 days after the delivery. The baby was given intravenous fluid drip of 3 cc/hour D10%, intravenous antibiotic consisting of 125 mg of ampicillin every 12 hours and gentamicin 10 mg every 24 hours, as well as 1 mg of vitamin K injection intramuscularly. The baby was then consulted to the senior pediatric surgeons for cloacal repair elective surgery consisting of ileostomy, caecal plate tubularization, and bladder closure.

## 3 Discussion

Cloacal exstrophy (CE) is a very complex anatomical anomaly and one of the most difficult congenital abnormalities to reconstruct. It is defined as ventral abdominal wall defect with omphalocele as well as bowel and bladder exstrophy. Omphalocele is located at the superior border, whereas the exposed ileocaecal area and bladder are located at the inferior border. A zone of intestinal mucosa separates the hemibladders at the midline, with a ureteral orifice at each hemibladder. The proximal intestinal opening often proliferates making an overall appearance resembling an elephant trunk. The distal bowel is a blind pouch. All babies born with CE have imperforate anus, and 85% of them have neural septal defects [4]. Hence, the name omphalocele-exstrophy-imperforate anus-spinal defects (OEIS) syndrome. All male babies have genital anomalies, including undescended testes and bifid penises [4,5].

Cloaca is divided into urogenital sinus anteriorly dan hindgut posteriorly at six weeks of pregnancy. The failure of lateral to medial mesoderm extension to form an infraumbilical abdominal wall causes cloacal membrane rupture. Cloacal exstrophy is caused by the rupture of the cloacal membrane before the complete descent of the urogenital septum, causing herniation of the bladder and bowel that can be detected using prenatal ultrasound [6].

With current advances in neonatal care and surgical techniques, the survival rate for CE can reach 100%. Thus, early prenatal diagnosis and transfer to a tertiary medical center for further neonatal care are essential for newborns with CE. However, early prenatal diagnosis of CE remains a challenge because of the complicated anatomy of the associated anomalies [7]. A correct prenatal diagnosis of CE is rarely made, even if pelvic or genitourinary abnormalities are suspected on prenatal ultrasound examination. In a study reviewing antenatal sonographic findings in patients born with CE, anomalies were detected in 54% (27/50) of cases but the correct antenatal diagnosis only occurred in 6% (3/50) of them. Most of the cases are misdiagnosed as hydronephrosis [8].

The diagnosis of CE should be suspected in the second or third trimester if there is ventral wall defect associated with persistent bladder non visualization in a fetus with normal amniotic fluid volume; genitalia identification difficulty beyond 20 weeks of gestational age; hemivertebrae with kyphoscoliosis, or evidence of lumbosacral meningomyelocele. The fetal bladder usually begins to fill again within a few minutes of urination; thus, a prolonged period of non visualization will indicate abnormalities. The prolapsed terminal ileum has a characteristic “elephant trunk” appearance. In the first trimester, a cystic mass can sometimes be identified, before the rupture of the cloacal membrane. There also appears to be an association between increased nuchal translucency and CE [6]. Accurate prenatal diagnosis of bladder exstrophy (BE) and CE is essential for appropriate prenatal counseling. The imaging sensitivity of MRI (83%) is better than ultrasound (69%) for determining the postnatal diagnosis. However, it is not feasible to use MRI as an early diagnostic tool for CE, especially in developing countries. The key feature to distinguish a prominent bladder plate in classic BE from omphalocele in CE is the location of the umbilical cord insertion in the abdominal wall in relation to the abdominal wall defect [9,10].

In an attempt to establish criteria for the early prenatal diagnosis of cloacal exstrophy, Meizner et al. (1995) reviewed sonographic findings in six CE cases and compared them with sonographic features of other anterior abdominal wall defects. Of the six cases, specific sonographic signs were observed: (1) large defect in the anterior midline infraumbilical line with prominent omphalocele; (2) absent bladder; (3) narrowed thorax; (4) distorted spine; (5) sacral myelomeningocele; and (6) bilateral club feet. In all cases, the bowel was noted to be floating in a large amount of ascitic fluid. Polyhydramnios is present in four out of six cases [11]. These sonographic features can assist the sonographer in differentiating CE from another midline anterior abdominal wall defects. MRI can be performed on a fetus with suspected CE, this is additional information to determine the anatomy of the pelvis that has anomaly and the potential for myelomeningocele [9].

In our case, the prenatal ultrasound at 33–34 weeks of pregnancy showed a defect in the midline of the anterior abdominal wall with prominent omphalocele and persistent bladder non visualization despite the normal amniotic fluid volume. The finding is in line with previous studies of CE cases. However, the diagnosis was made in the third semester, so the amount of time for prenatal counseling was limited. In Indonesia, it is not legal to terminate a viable fetus with major congenital anomalies. Nonetheless, prenatal counseling is important to prepare the parents to raise a child with a special trait. Early prenatal diagnosis is crucial not only for early management but also for the long-term quality of life of CE patients.

## 4 Conclusion

Omphalocele and persistent bladder non visualization despite normal amniotic fluid volume detected by prenatal ultrasound can be suggestive of CE. Accurate prenatal diagnosis of CE is important to carry out multidisciplinary management and prenatal counseling to parents.

## Provenance and peer review

Not commissioned, externally peer-reviewed.

## Sources of funding

The authors have no funding support to report.

## Ethical approval

Not applicable.

## Author contribution

F performed manuscript draft writing, conceptualization, and resources; F and MAA performed a critical review and editing of the manuscript. All authors have checked the final version of the manuscript.

## Registration of research studies

1. Name of the registry: N/A.
2. Unique Identifying number or registration ID: N/A.
3. Hyperlink to your specific registration (must be publicly accessible and will be checked): N/A.

## Guarantor

Febriani accepts full responsibility for the work and/or the conduct of the study, had access to the data, and controlled the decision to publish.

## Consent

Written informed consent was obtained from the patient's guardian for publication of this case report and accompanying images. A copy of the written consent is available for review by the Editor-in-Chief of this journal on request.

## Declaration of competing interest

The authors declared no conflict of interest.
